# LDHA-Mediated Glycolytic Metabolism in Nucleus Pulposus Cells Is a Potential Therapeutic Target for Intervertebral Disc Degeneration

**DOI:** 10.1155/2021/9914417

**Published:** 2021-06-10

**Authors:** Longxi Wu, Jieliang Shen, Xiaojun Zhang, Zhenming Hu

**Affiliations:** Department of Orthopedics, The First Affiliated Hospital, Chongqing Medical University, China

## Abstract

The intervertebral disc degeneration (IDD) is considered to be an initiator of a series of spinal diseases, among which changes in the nucleus pulposus (NP) are the most significant. NP cells reside in a microenvironment with a lack of blood vessels, hypoxia, and low glucose within the intervertebral disc. Due to the strong activity of HIF-1*α*, glycolysis is the main pathway for energy metabolism in NP cells. Our previous study found that higher SIRT1 expression is beneficial to delay the degeneration of NP cells. In order to find the downstream genes by which SIRT1 acts on NP cells, we used iTRAQ sequencing to detect the differences between degenerated NP cells overexpressing SIRT1 and a control group (human NP cells were derived from surgery) and found that the expression of LDHA changed in the same direction with SIRT1. This suggests that SIRT1 may delay the degeneration of NP cells by regulating glycolysis. We then used a Seahorse XFe24 analyzer to measure the bioenergetic parameters of NP cells and obtained three findings: (a) glycolysis is the main energy metabolism pathway in NP cells, (b) there is a large difference in ATP production between senescent cells and young cells, and (c) SIRT1 can regulate the production of ATP from glycolysis by regulating LDHA. We also found that SIRT1 in NP cells has a positive regulatory effect on c-Myc which is an upstream gene of LDHA. Through observing IDD-related indicators such as apoptosis, proliferation, senescence, and extracellular matrix, we found that SIRT1 can delay degeneration, and interference with c-Myc and LDHA, respectively, weakens the protective effect of SIRT1. Interfering with LDHA alone can also inhibit glycolysis and accelerate degeneration. Overall, we found that the inhibition of glycolysis in Np cells significantly affects their normal physiological functions and determined that LDHA is a potential therapeutic target for the treatment of IDD.

## 1. Introduction

Intervertebral disc degeneration IDD is considered by many doctors to cause a series of spinal diseases. Degenerative disc disease (DDD) caused by IDD is closely related to aging, stress, and other factors [1]. Problems related to aging in today's world are becoming more common. Due to the high incidence of DDD, high rate of disability, and high medical expenses, it has gradually become a serious public health problem, yet current clinical treatments for DDD are not satisfactory. Both medical treatment and physical therapy for the early-stage degeneration, or the removal, fusion, and replacement of the intervertebral disc for late stage degeneration, do not fundamentally restore the original biological characteristics of the intervertebral disc [2]. Spinal surgeons are faced with this treatment problem, but it remains unresolved.

Normal intervertebral disc tissue includes upper and lower cartilage endplates, surrounding fibrous annulus, and jelly-like NP tissue wrapped in the middle. Studies have found that the intervertebral disc mainly exchanges substances externally through penetration of the cartilage endplate [3]. This is because the blood vessels of the normal intervertebral disc are only distributed in the outer third of the annulus fibrosus, and the rest, especially the NP tissue, have no blood vessels. Such inadequate material exchange creates NP tissue in a harsh microenvironment with low oxygen and low nutrition [4]. Changes in the NP tissue are highly significant in degenerating intervertebral discs [5]. As the apoptosis of NP cells increases, their proliferation decreases, and their secretion of extracellular matrix decreases, which leads to changes in the structure and function of normal intervertebral discs and decreases their biomechanical properties [6].

Carbohydrate metabolism is the main energy source for most cells, as well as in NP cells. In previous studies, NP cells observed via electron microscopy were found to contain complete mitochondria, but mitochondria numbers were significantly reduced compared with other cells [7]. Some scholars have found that hypoxia-inducible factor (HIF-1*α*) is highly expressed in the NP to inhibit oxidative phosphorylation, so it may be that NP cells have adapted to a hypoxic environment [8]. Glycolysis is considered to be the main carbohydrate metabolism pathway of normal NP cells [9]. Compared with oxidative phosphorylation, glycolysis is a fast but inefficient process to produce ATP, so the energy production of most cells is dominated by oxidative phosphorylation. However, immune cells and cancer cells, for example, even in an aerobic environment, still mainly use glycolysis to produce ATP (aerobic glycolysis) [10]. This mode of carbohydrate metabolism not only plays an important role in maintaining energy homeostasis but also may be crucial to signal transduction that alters cell function [11–13]. Changing this metabolic homeostasis may have a large impact on the normal functioning of NP cells, and our work is based on this hypothesis.

In this study, we hypothesized that the decrease of SIRT1 expression in NP cells affects normal energy metabolism and that changes in their metabolic mode may lead to abnormal physiological functions and aggravate degeneration. In this work, tissue samples were obtained from intervertebral discs removed during operations on patients with lumbar vertebral fractures (LVF, Pfirrmann I–II) and intervertebral disc degeneration (IDD, Pfirrmann IV–V). The iTRAQ sequencing results showed that LDHA is located downstream of SIRT1, so SIRT1 may maintain energy metabolism by regulating glycolysis. We then used the Seahorse XFe24 detector to find that SIRT1 can regulate glycolysis-based energy metabolism in NP cells through LDHA. We also found that SIRT1 may regulate LDHA through c-Myc, which is upstream of LDHA. Our findings reveal a new way of regulating energy metabolism in NP cells and provide new ideas for the treatment of IDD.

## 2. Materials and Methods

### 2.1. NP Tissue Collection

The NP tissue in this study came from the spinal surgery patients admitted to the second group of orthopedic spine of the First Affiliated Hospital of Chongqing Medical University. We use the Pfirrmann grading system to determine the degradation level of IVD through preoperative MRI scans. Patients with vertebral fractures and herniated discs with a score of I-II are defined as nondegeneration or early degeneration, and discs with a score of IV-V are defined as the advanced stage of IVDD, such as elderly patients. All experimental samples were taken from patients undergoing lumbar spine surgery (average age: 40 years old; age range: 18-70 years old; PfirrmannI-II: 5 males and 5 females; and PfirrmannIV-V: 5 males and 6 females). This study has been approved by the Ethics Committee of Chongqing Medical University, and all patients participating in this study have obtained informed consent.

### 2.2. Isolation and Culture of Primary NP Cells

We have long been engaged in the related research of NP. The isolation and culture of primary NP cells from surgical patients are as follows. First, after obtaining the NP tissue during the operation, it should be transported to the experimental environment on wet ice for no more than 2 hours. In a sterile environment, we use surgical instruments such as forceps and ophthalmic scissors to separate the NP from the tissue; remove the annulus fibrosus, endplates, and vertebrae; and cut into the smallest possible flocs. Then, the collected tissue pieces were digested with 0.25% pancreatin solution (Sigma, USA) at 37°C for 1 hour. After removing the pancreatin solution, they were digested with 0.2% type II collagenase (Sigma, USA) for about 4 hours. After that, we use a complete medium (DMEM/F-12 (Gibco, United States) containing 15%-20% fetal bovine serum (FBS, Gibco, United States)) to neutralize type II collagenase (it is recommended not to add penicillin to complete medium)/streptomycin). Then, the filtrate passed through a 200-mesh filter was centrifuged at 1200 rpm for 10 minutes to precipitate NP cells. Then, the cells were resuspended in complete medium and inoculated into a 5 ml perforated culture flask and placed in an incubator (37°C, 5% CO_2_). For the first inoculation, let it stand for 7 days to allow as many NP cells to grow adherently. When the cell confluence reached 80-90%, NP cells were trypsinized with 0.25% trypsin (Sigma, USA) for subculture. All cell experiments use the second- to third-generation NP cells.

### 2.3. NP Cell Transfection

Recombinant lentiviral vectors (Ad-SIRT1, SIRT1-shRNA, and LDHA-shRNA) were purchased from GeneChem (Shanghai, China). According to user instructions, NP cell transfection is carried out. Through preliminary experiments, the NP infection coefficient (MOI) of this batch of virus is about 15. We add the lentiviral vector when the NP cells reach 30-50% confluence. Observing after 12 hours, if the cells are in good condition, we replace the medium. After three days, we observe the fluorescence to judge the transfection efficiency and replace it with a complete medium containing 2 *μ*g/ml puromycin. We continue to culture for 3-5 days. After the transfection efficiency reaches 90% or more, we use WB or PCR to judge whether the transfection is successful and then use it for subsequent experiments.

### 2.4. SA-*β*-gal Staining

SA-*β*-gal staining is used to detect the degree of senescence of NP cells, and the operation flow is in accordance with the instructions of the SA-*β*-Gal kit (Beyotime, China). In short, NP cells (1 × 10^6^/well/group) cultured in a 6-well plate were washed twice with PBS buffer and then fixed with 0.2% paraformaldehyde for 20 minutes at room temperature. Then, the cells to be tested were stained with X-gal solution overnight (37°C, no CO_2_). Images were taken by an optical microscope (Olympus, Japan), and the percentage of SA-*β*-gal-positive cells was counted for statistical analysis.

### 2.5. Immunohistochemical Staining

Immunohistochemical staining is used to evaluate the protein deposition and expression levels of SIRT1, LDHA, C-Myc, and ECM in NP tissue and NP cells. In short, the NP tissue obtained by surgery was fixed with paraformaldehyde for 24 hours, embedded in paraffin, and cut into 4 mm sections. Then, the sections were treated with %3 H_2_O_2_ at room temperature for 15 minutes, followed by incubation with 0.125% trypsin at 37°C for 30 minutes, and then blocked with normal goat serum for 15 minutes. The sections were incubated with different rabbit antibodies at 4°C overnight. Then, the sections were incubated with goat anti-rabbit IgG-HRP secondary antibody (1 : 1000; Proteintech, China) and then counterstained with hematoxylin. After completing the process, we take an image (200x) with an optical microscope (Olympus, Japan). We count the five areas (200 times) across the entire section, calculate the positive rate, and perform statistical analysis.

### 2.6. Immunofluorescence Staining

The NP cells were seeded in a 24-well plate with cell slides. After the cells reached the target confluence, the slides were taken out and washed with PBS buffer three times. Then, the cell slide was immersed in 4% paraformaldehyde for 10 minutes to fix the cells and then treated with 0.5% Triton for 5 minutes. Then, the cell slide was covered with different rabbit antibodies and kept at 4°C overnight. Next, we incubate with anti-rabbit fluorescent secondary antibody (1 : 1000; Proteintech, China) for 2 hours at room temperature in the dark. The cells were then counterstained with 4′,6-diamidino-2-phenylindole (DAPI), and images were collected using a fluorescence microscope (Leica, Germany).

### 2.7. Flow Cytometer

We use the Annexin V/PI apoptosis detection kit (LIANKE, Hangzhou, China) to detect apoptotic cells. In short, the NP cells (1 × 10^6^/well/group) cultured in a six-well plate were collected, washed twice with PBS buffer solution, resuspended in the binding fluid, and mixed with 5 *μ*l Annexin V-FITC, and 20 *μ*l PI (Hanbio (Shanghai, China)) was placed in the dark at room temperature for 15 minutes. Finally, 400 *μ*l of binding buffer was added to the cell sample. Immediately after staining, the cells were analyzed by flow cytometry. The apoptosis rate is determined by the sum of the percentages of early (Annexin V +/PI-) and late apoptotic cells (Annexin V +/PI +). When measuring the cell cycle of the NP, the NP cells cultured in a six-well plate (1 × 10^6^/well/group) were collected and then washed twice with PBS buffer solution. The cells were fixed with prechilled 75% ethanol and stored at 4°C overnight. The cells are incubated with propidium iodide solution and then measured by flow cytometry.

### 2.8. EdU Cell Proliferation Detection

EdU cell proliferation detection kit (Beyotime, China) was used to detect the proliferation of NP cells. The NP cells were seeded in a 24-well plate with cell slides. After the cells reached the target confluence, the preheated EdU working solution was added to the culture medium, and the incubation continued for 4 hours. Then, the cell slide was taken out, fixed with 4% paraformaldehyde for 15 minutes at room temperature, washed with PBS solution containing 3% BSA, and then treated with 0.3% Triton for 10 minutes. We incubate the cell slide with click additive solution for 30 minutes in the dark at room temperature. Then, the nuclei were stained with Hoechst, and images were collected using a fluorescence microscope (Leica, Germany).

### 2.9. Protein Extraction and WB

The NP cells washed three times with PBS were lysed with 1% PMSF-containing RIPA lysis buffer (Beyotime, China) at 4°C for 30 minutes and then centrifuged at 12000 × g for 15 minutes at 4°C, and the supernatant was collected. We use enhanced bicinchoninic acid (BCA) kit (Beyotime, China) to detect the protein concentration of the sample. Then, the obtained sample was subjected to 8-12.5% SDS-polyacrylamide gel electrophoresis (SDS-PAGE) and transferred to a PVDF membrane (Millipore, USA) by electroblotting. The PVDF strips were blocked in TBST (TBS with 1% Tween 20) containing 5% blocked milk powder for 1 hour at room temperature. Then, they were incubated with different primary antibodies at 4°C overnight. After washing the strips with TBST three times, they were incubated with goat anti-rabbit secondary antibody (1 : 1000, Proteintech, China) for 1.5 hours at room temperature. After ECL imaging, the intensity of the blot was analyzed by Image Lab software (Bio-Rad, USA).

### 2.10. iTRAQ

The iTRAQ testing for this work was commissioned by BGI (Shenzhen, China) for testing. In short, what we did was to construct the NP cells of the SIRT1 overexpression group and the control group. After the NP cells were successfully transfected, WB technology was used to verify the success of SIRT1 overexpression. We collect four copies of the NP cells in the treatment group and the control group of 10 cm culture dishes (test pair *n* = 3, backup pair *n* = 1). After quick freezing in liquid nitrogen, they are stored in dry ice and sent to the testing laboratory. After receiving the sample, the BGI laboratory has passed the quality assessment of the sample and then undergoes proteolysis, peptide labeling, peptide separation, LC-MS/MS, and other steps to complete the iTRAQ test. We use Dr.Tom Mass Spectrometry Cloud System (BGI, China) to perform protein difference analysis, differential protein GO analysis, and differential protein pathway enrichment on experimental reports.

### 2.11. Seahorse Testing

The NP cells of each treatment group and the control group were seeded on Seahorse XFe24 plates at a rate of 5000 per well, and after three days of normal culture, the Seahorse test was performed. We prepare the required reagents and consumables one day before the experiment according to the Seahorse XF Real-Time ATP Rate Assay (Agilent, USA) instructions and preheat the Seahorse XFe24 detector (Agilent, USA) overnight. A formal experiment was carried out the next day. After running the machine into the cell plate, we add oligomycin and rotenone/antimycin A in turn and record the ECAR value and OCR value every five minutes. Wave software (Agilent, USA) was used to analyze the data.

### 2.12. Statistical Analysis

Unless otherwise specified, all quantitative tests were performed in triplicate, and the data were expressed as mean ± standard deviation. We use SPSS 20.0 software (SPSS, Inc., USA). We use GraphPad Prism 8 to perform statistical analysis using appropriate statistical tests and use Prism to generate graphs. A one-way analysis of variance (ANOVA, *P* < 0.05) was used to determine the statistically significant differences between the groups. Unpaired *t*-test (*P* < 0.05) was used to analyze individual groups. For the collocation between the control groups, the culture environment, the number of cultures, and the gender of the donor were considered.

## 3. Results

### 3.1. Decreased Expression of SIRT1 in Degenerative NP Tissue

**Figure 1:**
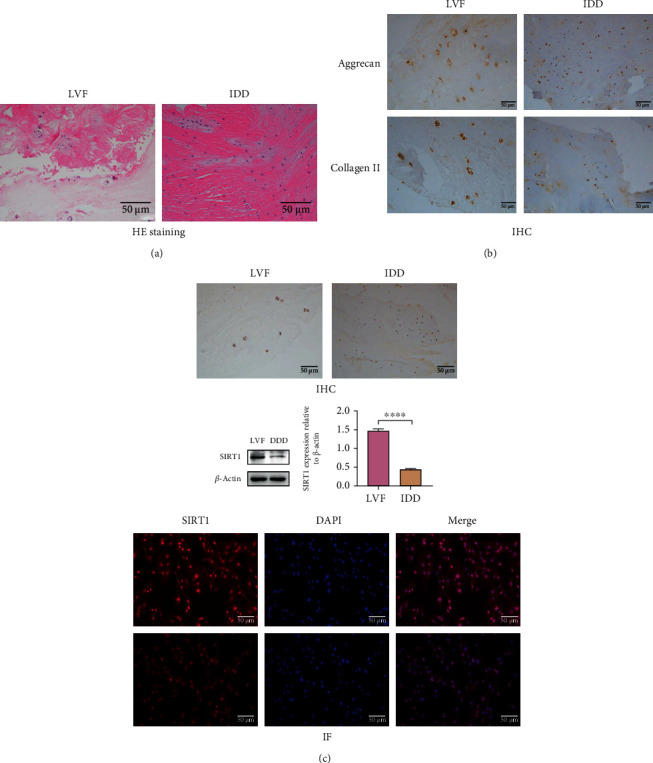
Preoperative lumbar MRI images of patient's intervertebral disc tissue were classified via Pfirrmann's grading system. Intervertebral disc tissues scored I-II were defined as normal disc or early stage of IDD, and the discs scored IV-V were defined as advanced stage of IDD (LVF: Pfirrmann I-II, IDD: Pfirrmann IV-V). Red arrows refer to the position of the acquired NP tissue. (a) HE staining (200x) showed that the volume of nucleus pulposus cells in IDD tissues decreased, and the cell spacing was reduced due to tissue shrink age. (b) Immunohistochemical staining (200x) shows the expression of Aggrecan and collagen II expression in the NP tissue from LVF and IDD patients. (c) IF and IHC showed that the expression of SIRT1 in severely degenerated NP cells was significantly reduced. Western blots and quantification data of SIRT1 in NP cells of each group. ^∗^*P* < 0.0001. Black and white bars = 50 *μ*m.

**Figure 2:**
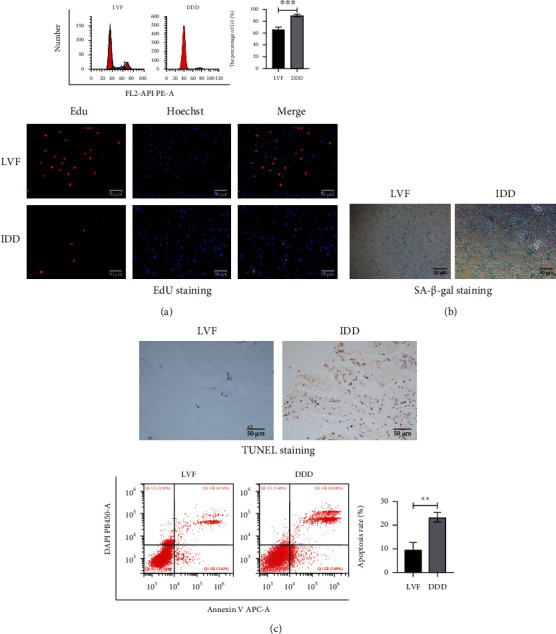
(a–c) SA-*β*-gal staining (200x), EdU staining (200x), and flow cytometry assay all showed that the IDD group had a higher degree of degeneration and worse normal physiological functions than the LVF group. ^∗^*P* < 0.0001. Black and white bars = 50 *μ*m.

First, we tested whether there was a relationship between SIRT1 expression in NP cells and disc degeneration. According to the Pfirrmann grading, the degree of degeneration of lumbar intervertebral discs in patients undergoing spinal surgery was evaluated. We classified patients by Pfirrmann grading and their condition, in order to obtain tissue samples with different degrees of degeneration. We used HE staining and immunohistochemical staining of the extracellular matrix to determine whether the tissue samples were NP tissues (Figure 1(a)). In the NP tissue and the primary cells extracted from it, the expression of SIRT1 in degenerative NP tissues was significantly lower than that of control samples (Figure 1(c)). NP degeneration is often accompanied by changes in indicators such as cell proliferation, apoptosis, senescence, and extracellular matrix. The results obtained by different detection methods showed that the proliferation of degenerated NP cells decreased, apoptosis increased, and aging increased (Figures 2(a)–2(c)). These results indicate that as the degeneration of the intervertebral disc progresses, the expression of SIRT1 decreases.

### 3.2. Decreasing SIRT1 Expression Aggravates Intervertebral Disc Degeneration

**Figure 3:**
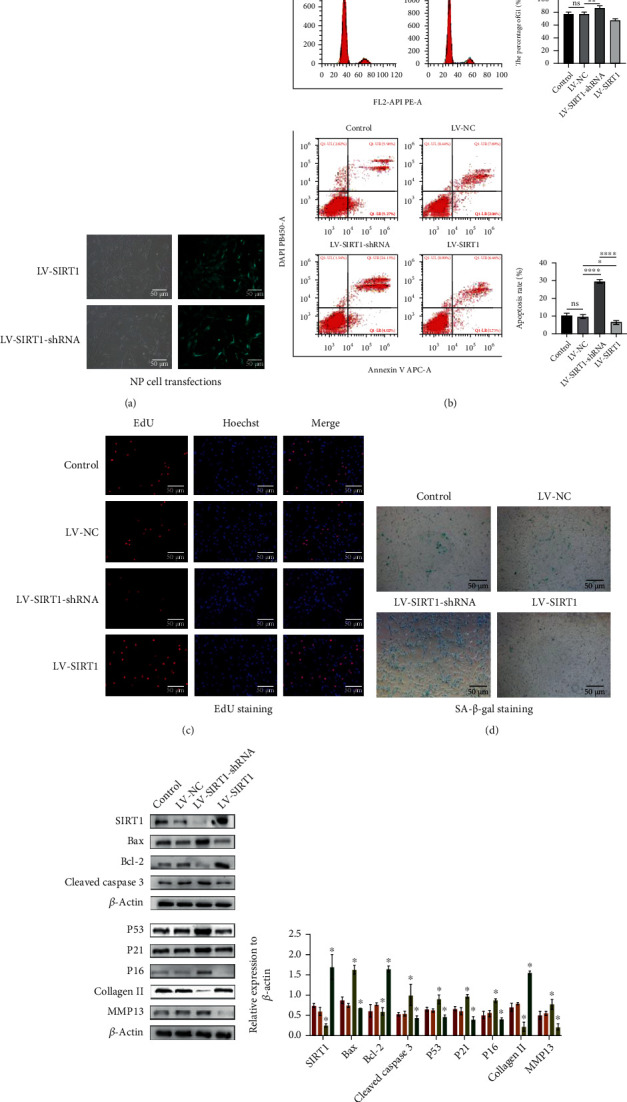
Decrease of SIRT1 expression will aggravate the progress of intervertebral disc degeneration. (a) The transfection efficiency of nucleus pulposus cells was over 80%，MOI = 15, puromycin concentration = 2 *μ*g/ml. (b) Flow cytometry assay showed that reducing the expression level of SITRT1 resulted in a decrease in the proliferation rate and an increase in the apoptosis rate. ^∗^*P* < 0.005. (c) EdU cell proliferation assay (200x). (d) Reducing the expression level of SIRT1 leads to accelerated senescence of NP cells (SA-*β*-gal staining (200x)).(e) The SIRT1, Bax, Bcl-2, cleaved caspase 3, and senescence-related protein (P53, P21, and P16) levels were analyzed by western blotting. ^∗^*P* < 0.05 versus the control group. Black and white bars = 50 *μ*m.

Although our results indicated that SIRT1 is low in degenerative NP cells, this does not mean that SIRT1 affects NP cell the degeneration. Therefore, we used a customized lentivirus to upregulate and downregulate SIRT1 (Figure 3(a)). Protein expression levels were detected by western blotting, and the results showed that SIRT1 was successfully upregulated or downregulated. Similarly, NP cells after successfully changing the SIRT1 expression were tested for degeneration-related indicators. The results showed that as the expression of SIRT1 was changed, the indexes related to the degeneration of NP cells changed to different degrees (Figures 3(b)–3(e)). In general, lower SIRT1 expression leads to less proliferation and extracellular matrix and more apoptosis and senescence.

### 3.3. Upregulation of SIRT1 Promotes Glycolysis

**Figure 4:**
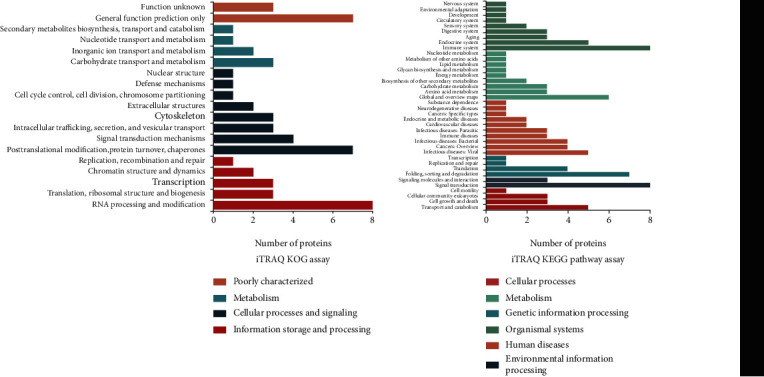
After the results of iTRAQ are enriched by KOG and KEGG, the differential proteins and differential pathways are counted (Dr.Tom, BGI, China). Activating LDHA can regulate energy metabolism to delay disc degeneration.

**Figure 5:**
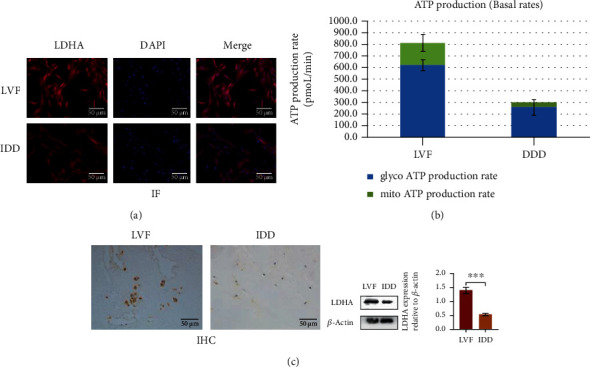
(a, c) LDHA in tissues and cells of LVF group was significantly higher than that in the IDD group. Western blots and quantification data of LDHA in NP cells of each group. ^∗^*P* < 0.05. Black bars = 50 *μ*m. (b) Both LVF and IDD nucleus pulposus cells have glycolysis as the main breathing mode, and the ATP production of the LVF group is significantly higher than that of the IDD group (Seahorse XF Real-Time ATP Rate Assay Kit). All data had been normalized by protein concentration of each well.

Regarding the mechanism of SIRT1 in the intervertebral disc, previous studies have reported that SIRT1 can delay IDD through autophagy-related pathways, while the effects of other pathways have not been reported. We used proteomics to detect NP cells. In order to avoid the problems of nonobvious differences in young NP cells and imperfect physiological functions of severely degenerated NP cells, we constructed a lentiviral overexpression SIRT1 cell model using moderately degenerated NP cells. After the results of the iTRAQ test were subjected to KOG and KEGG pathway analyses, we found that hundreds of proteins changed significantly with the overexpression of SIRT1 (Figure 4). Among them, in addition to proteins that had little correlation and those that have already been reported, we found that some proteins were concentrated in pathways related to sugar metabolism. One that was clearly different and is highly significant is LDHA. LDHA is the key enzyme of glycolysis, and glycolysis is the most important means of energy metabolism in the NP. Based on this, we found that the expression of LDHA in young NP cells was significantly higher than that in degenerative NP cells (Figures 5(a) and 5(c)). In addition, using the Seahorse XFe24 detector, we found that the real-time ATP production of degenerated NP cells was significantly reduced, and whether degenerated or not, the NP cells mainly used glycolysis (Figure 5(b)). These results suggest that SIRT1 can positively regulate glycolysis, which is most likely accomplished by regulating LDHA.

**Figure 6:**
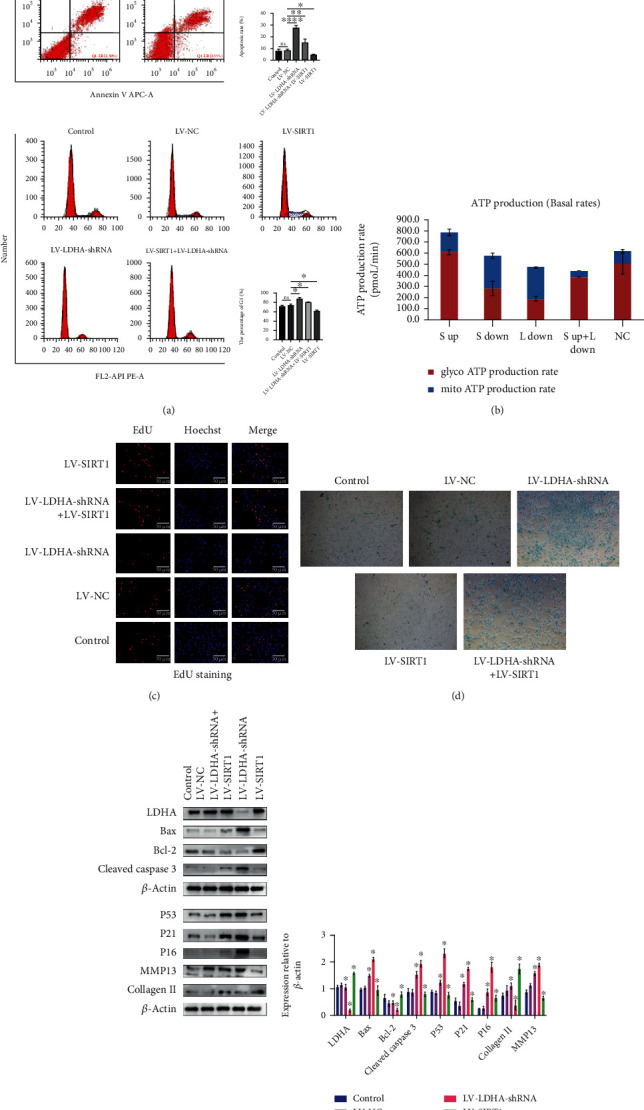
(a) Flow cytometry assay showed that regulating the expression level of SITRT1 and LDHA resulted in the proliferation rate and an in the apoptosis rate. ^∗^*P* < 0.005. (b) Seahorse detects the real-time ATP production of nucleus pulposus cells in different groups (S: SIRT1; L: LDHA). (c) EdU cell proliferation assay (200x).(d) SA-*β*-gal staining assay (200x). (e) The SIRT1, Bax, Bcl-2, cleaved caspase 3, and senescence-related protein (P53, P21, and P16) levels were analyzed by western blotting. ^∗^*P* < 0.05 versus the control group. Black and white bars = 50 *μ*m.

Next, in order to determine if SIRT1 is indeed regulating glycolysis through LDHA, we need to test whether that interference with LDHA can cause similar results as with interference with SIRT1, and if overexpression of SIRT1 after interference with LDHA can reduce that impact. In addition to the SIRT1 lentivirus used in the previous experiment, we used newly purchased LDHA-shRNA lentivirus. After cells were transfected with the lentivirus, puromycin was used to select stable strains. NP cells transfected with interfering LDHA + overexpressing SIRT1 lentivirus had to be transfected twice, as transfection with two viruses at the same time will result in the inability to survive due to high virus concentrations. After the cell model was constructed, flow cytometry analysis and EdU detection showed that the proliferation of NP cells decreased due to the interference of LDHA (Figures 6(a) and 6(c)). Western blot and flow cytometry analysis showed increased apoptosis of NP cells with the interference of LDHA (Figures 6(a) and 6(e)). SA-*β*-gal staining can be used to detect the degree of senescence of NP cells. The darker and higher the ratio of blue staining, the higher the degree of senescence. SA-*β*-gal staining and western blot analysis showed that with the interference of LDHA, the degree of senescence of NP cells increased (Figures 6(d) and 6(e)). Analysis by the Seahorse XFe24 detector showed that the interference with LDHA significantly reduced the amount of ATP produced by glycolysis, and the total ATP production was also affected (Figure 6(b)). In addition, the extracellular matrix content also decreased (Figure 6(e)). Importantly, the overexpression of SIRT1 reversed the results of LDHA interference. Taken together, SIRT1 is located upstream of LDHA, and by regulating LDHA, it can affect the carbohydrate metabolism of NP cells and thus affect the degeneration process. However, it has not been clarified whether SIRT1 has a direct effect on LDHA, and it is also unclear whether there is a bridge between them.

### 3.4. SIRT1 Positively Regulates LDHA through c-Myc

**Figure 7:**
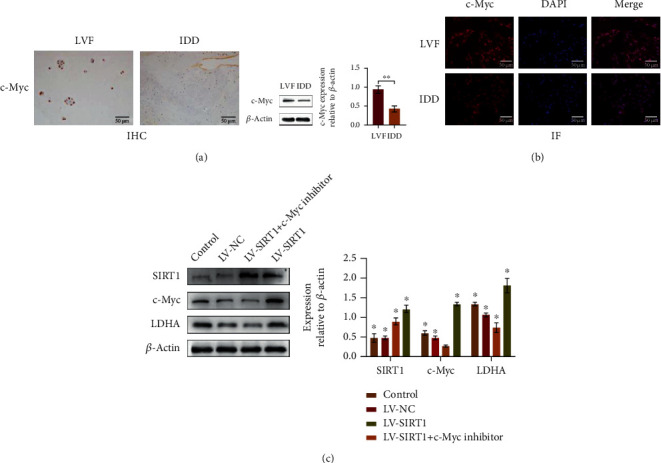
(a, b) LDHA in tissues and cells of the LVF group was significantly higher than that in the IDD group. Western blots and quantification data of LDHA in NP cells of each group. ^∗^*P* < 0.05. Black bars = 50 *μ*m. (c) Western blots showed that SIRT1 can regulate LDHA through c-Myc.

Studies have shown that SIRT1 may have a direct deacetylation effect on LDHA, but there is no evidence that deacetylation can change the function and expression level of LDHA. We predicted through the protein interaction network that the c-Myc protein, which is closely related to both SIRT1 and LDHA, may play an important role. As a nuclear protein that can regulate cell growth and division, c-Myc can activate SIRT1 expression to inhibit aging and death, and SIRT1 can counteract c-Myc to form a negative feedback. In normal cells, the two coexist in an orderly manner and are jointly regulated by a variety of other factors. In cancer cells, dysregulated c-Myc and SIRT1 promote an indefinite cell. LDHA is a downstream gene of C-Myc, and the Warburg-like effect of NP cells suggests its involvement. We used western blotting and immune cell fluorescence to detect the c-Myc expression levels of normal and senescent NP cells and found that young NP cells had significantly higher c-Myc than senescent NP cells (Figure 7(a)). An immunohistochemical comparison of young and aging tissues also yielded consistent results (Figure 7(b)). Next, we added a c-Myc inhibitor to the culture of a stable NP cell strain overexpressing SIRT1 and found that the expression level of LDHA was significantly lower than that of control cells without the inhibitor (Figure 7(c)). Therefore, we determined that SIRT1 can increase the expression level of downstream effectors through positive regulation by c-Myc, thereby promoting the downstream gene LDHA to improve glycolysis.

## 4. Discussion

**Figure 8:**
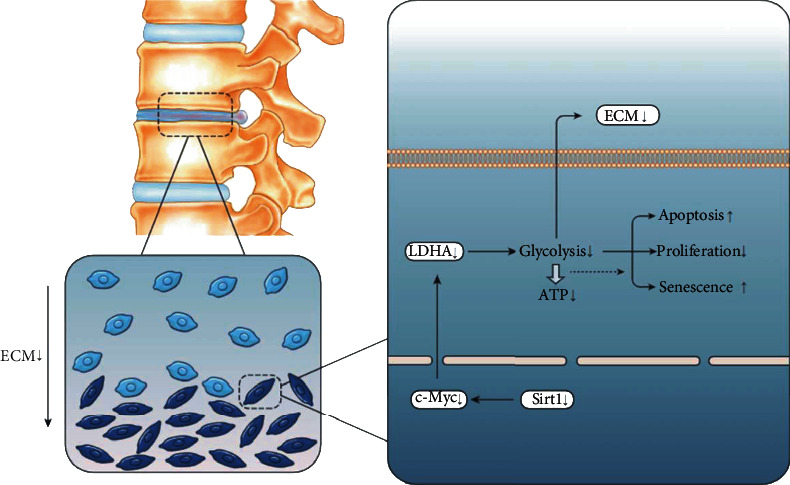
Schematic diagram shows potential mechanism of action of SIRT1-LDHA.

The findings in this work help clarify the relationship between the causes of IDD and energy metabolism and reveal potential therapeutic. We came to the following conclusions: (a) SIRT1 delays the degeneration of the intervertebral disc. (b) In contrast to previous studies on SIRT1, its positive function on NP cells is mediated partly through energy metabolism (glycolytic enzymes) controlled by LDHA. (c) c-Myc acts as a bridge between SIRT1 and LDHA. In general, a high expression level of SIRT1 means a high expression level of LDHA, which means that more glycolysis generates more ATP for physiological activities. This avoids the negative effects of incomplete oxidative phosphorylation on NP cells. Therefore, LDHA can be used as a potential therapeutic target to delay the degeneration of NP cells. Schematic diagram shows potential mechanism of action of SIRT1-LDHA (Figure 8).

In contrast to other forms of cartilage, the NP has several structural layers, the environment is more severe, and the stress it receives is the highest among all cartilage, so the reasons for its degeneration must be more complicated [14]. All cells rely on energy to complete normal physiological activities. Here, we provide evidence that glycolysis-related energy metabolism disorders are a main driving force of pathological changes in NP degeneration. During the lifetime of the NP, genes with positive effects such as LDHA gradually decrease in expression, which is due to continuous loss caused by the harsh environment. For example, mechanical stress, material exchange, and aging, and even various normal functions that maintain the normal work of cells become disordered [15]. Greater degeneration of the extracellular matrix leads to changes in the normal morphology of the intervertebral disc, its height decreases, and the water content decreases, which leads to a further increase in mechanical stress to the intervertebral disc damage. As a result, the already difficult material exchange becomes worse, creating a vicious circle.

However, due to the special material exchange of the NP and its harsh environment, it is necessary to consider the use of LDHA activators on the human body. It is not easy to reach a dose concentration locally, and systemic energy metabolism disorders are possible. Studies have shown that people with nonfunctional LDHA mutations are usually asymptomatic or mildly symptomatic after strenuous exercise, which indicates that the regulation of LDHA may not have obviously harmful effects on healthy cells [16, 17]. If LDHA as a target is very safe for humans, this work will have greater significance. However, even if an effective drug was found, a unique method of administration would be needed. There is no space inside the intervertebral disc, and invasive treatment methods such as direct injection also have problems with poor local aggregation and slow drug diffusion. New treatment may be adopted, such as IL-1*β* treatment to induce metabolic reprogramming, mimicking the Warburg effect, which has been confirmed in some previous reports on cartilage cell metabolism [18–20]. However, this will be more difficult to administer to senescent NP cells. As degeneration intensifies, the height of the intervertebral disc decreases, the water content decreases, and normal physiological functions are gradually lost, making it more difficult for external drugs to enter the NP [21]. Even if a drug successfully reaches the NP, the function of LDHA is restored, ATP production increases, and NP function is partially restored, the problem of lactic acid accumulation still exists. Lactic acid is a product of glycolysis, and studies have shown that too high a concentration can affect the growth of NP cells [22]. So why is it that young NP cells produce more lactic acid but are not affected by high concentrations of lactic acid? We believe this is because of changes in the extracellular environment, i.e., the extracellular matrix. Although the lactic acid production of degenerated NP cells is lower than that of normal NP cells, glycolysis is still the main source of ATP. As the degeneration intensifies, the already poor material exchange system becomes increasingly stretched. In other words, normal NP cells produce a large amount of lactic acid and can efficiently remove it, but the degenerated NP cells cannot do so, and over time, they accumulate excessive lactic acid. At present, there are ideas for rehydration and lactic acid removal in the NP, such as partial expansion and decompression and particulate material encapsulation of drugs. The development of these technologies will promote progress on NP treatment.

We have also observed that by reducing the expression level of LDHA, NP cells will compensate by increasing the ATP produced by oxidative phosphorylation over the short term due to lack of glycolysis. We speculate that this may be another reason for the degeneration of NP cells. Although the cells have mitochondria, the number is limited, which causes electron leakage during incomplete oxidative phosphorylation and produces a large number of oxygen free radicals [23]. The oxidative stress caused by the accumulation of reactive oxygen species (ROS) causes the senescence of NP cells and damage the synthesis of extracellular matrix [24]. In this system, as an antioxidative stress molecule, SIRT1 has been confirmed to play a role in ROS resistance. Interestingly, a brief increase in ROS may lead to the production of SIRT1, which in turn leads to a decrease in ROS [25]. Current research indicates a complex signaling network including SIRT1/FOXO, SIRT1/NF-*κ*B, SIRT1/NOX, SIRT1/SOD, and SIRT1/eNOs, which are mediated by SIRT1 and reduce ROS [26]. The role of ROS in this regulatory mechanism will be the focus of our follow-up work. In addition, SIRT1 may have a direct effect on LDHA in addition to the mutual promotion of LDHA with c-Myc [27–29]. The longevity regulator SIRT1 is a NAD+-dependent deacetylase. Studies have shown that LDHA can be directly deacetylated by SIRT1, but it does not seem to directly affect the expression and activity of LDHA. However, acetylated proteins will increase the risk of degradation by autophagy mediated by the molecular chaperone pathway, so the deacetylation of SIRT1 may indirectly protect LDHA.

We found evidence of an energy regulatory mechanism of SIRT1-c-Myc-LDHA and propose a new role for SIRT1 in protecting NP cells. In addition, this discovery also promotes our understanding of chronic orthopedic diseases such as IDD. Excessive damage and incomplete repair are important, but the accumulation of normal physiological functions like energy metabolism cannot be ignored. One limitation of this study is the inability to use fluorescent probes such as DCFDA to study the role of ROS in this mechanism. In addition, the lack of appropriate experimental animal models for IDD limits *in vivo* experiments. The focus of our future work will be to regulate LDHA-related glycolysis for clinical treatment, find and establish an experimental animal platform that is as close to humans as possible, and use technologies such as redox sensors to perform more detailed analysis of ROS types [30]. In summary, our results confirm that LDHA-related glycolysis regulation is a new therapeutic target for IDD.

## Data Availability

The data used to support the findings of this study are available from the corresponding author upon request.
